# Role of L2 cysteines in papillomavirus infection and neutralization

**DOI:** 10.1186/1743-422X-6-176

**Published:** 2009-10-27

**Authors:** Ratish Gambhira, Subhashini Jagu, Balasubramanyam Karanam, Patricia M Day, Richard Roden

**Affiliations:** 1 Department of Pathology, The Johns Hopkins University, Baltimore, MD 21231, USA; 2 Department of Oncology, The Johns Hopkins University, Baltimore, MD 21231, USA; 3 Department of Obstetrics and Gynecology, The Johns Hopkins University, Baltimore, MD 21231, USA; 4 Tulane National Primate Research Center, 18703, Three Rivers Road, Covington, LA 70433, USA; 5 National Cancer Institute, Bethesda, MD 20892, USA

## Abstract

Vaccination of mice with minor capsid protein L2 or passive transfer with the L2-specific neutralizing monoclonal antibody RG-1 protects against human papillomavirus type 16 (HPV16) challenge. Here we explored the nature of the RG-1 epitope and its contribution to viral infectivity. RG-1 bound equivalently HPV16 L2 residues 17-36 with or without an intact C22-C28 disulphide bridge. HPV16 L2 mutations K20A, C22A, C22S, C28A, C28S, or P29A prevented RG-1 binding, whereas Y19A, K23A or Q24A had no impact. Mutation of either C22 or C28 to alanine or serine compromises HPV16 pseudoviral infectivity both *in vitro *and in the murine vaginal tract, but does not impact pseudovirion assembly. Despite their lack of infectivity, HPV16 pseudovirions containing C22S or C28S mutant L2 bind to cell surfaces, are taken up, and expose the 17-36 region on the virion surface as for wild type HPV16 pseudovirions suggesting normal furin cleavage of L2. Mutation of the second cysteine residue in Bovine papillomavirus type 1 (BPV1) L2 to serine (C25S) dramatically reduced the infectivity of BPV1 pseudovirions. Surprisingly, in contrast to the double mutation in HPV16 L2, the BPV1 L2 C19S, C25S double mutation reduced BPV1 pseudovirion infectivity of 293TT cells by only half.

## Findings

Papillomavirus infection requires cleavage of minor capsid protein L2 by furin [1]. Mature virions in solution are resistant to furin cleavage and RG-1 binding [2-4]. The binding of virions to cell surfaces, presumably via heparan sulfate proteoglycans [5], promotes furin cleavage of L2, and this can occur on the cell surface. Furin cleavage triggers a conformational change that improves the accessibility of L2 on the capsid surface and its recognition by RG-1 [4]. RG-1 recognizes L2 residues 17-36 [2], and vaccination with this peptide in the appropriate context triggers high titers of neutralizing antibodies and protection against experimental challenge with homologous as well as heterologous virus types [6]. The cross-protective nature of this L2 epitope is consistent with its high degree of sequence conservation among diverse papillomavirus genotypes, and may reflect evolutionary constraints due to critical biological functions within this region [7]. Therefore, we sought to identify L2 residues critical to papillomavirus biology by deletion and alanine scanning mutagenesis within the epitope defined by RG-1. The role of L2 in infection is conserved in diverse papillomavirus types [8], but here we focus upon HPV16 because it is associated with a half of cervical cancer cases and the majority of HPV+vaginal, vulval, penile, anal, and head and neck cancers [9].

Sequences of the codon-modified HPV16 L2 gene within the region encoding the RG-1 epitope were deleted to generate the Δ17-30 and Δ23-36 deletion mutants [10]. As controls, two additional deletion mutants Δ353-362 and Δ393-403 were prepared with similarly sized deletions introduced at the C-terminus of HPV16 L2. The four deletion mutants or wild type HPV16 L2 were co-transfected into 293TT cells with an HPV16 L1 expression vector [10] and the SEAP reporter plasmid [11,12]. Three days later the cells were harvested and detergent lysates were treated with benzonase to remove unencapsidated DNA. HPV16 pseudovirions were purified using standard protocols ([11,12] as detailed in ). By comparison with wild type HPV16 L2, the introduction of these deletions within L2 had no significant impact upon the yield of particles in the appropriate gradient fraction, as demonstrated by L1 Western blot analysis. Likewise, Western blot analysis of purified HPV16 pseudovirions revealed similar levels of wild type and deletion mutant HPV16 L2 were present, suggesting that none of the small deletions within L2 adversely impacted L1/L2 co-assembly into particles. Extraction of benzonase-resistant, and therefore presumably encapsidated [11], DNA from the purified HPV16 pseudovirions and visualization by agarose gel electrophoresis revealed that L2 wild type and mutant particles contained similar levels of encapsidated reporter plasmid, implying that none of these small deletions within L2 prevented DNA encapsidation. HPV16 pseudovirions prepared in the absence of L2 were not infectious above 0.1% of those containing wild type L2, as demonstrated by measuring the ability to deliver the SEAP reporter plasmid to 293TT cells (Table 1). HPV16 pseudovirions carrying the L2 Δ17-30 and Δ23-36 had no detectable activity (i.e. >0.1% of wild type), whereas the C-terminal deletion mutants Δ353-362 and Δ393-403 exhibited similar activity to wild type L2.

**Table 1:** Assembly, infectivity and RG-1 antibody reactivity of pseudovirions carrying mutant L2

**Mutation within HPV16 L2**	**Infectivity of mutant HPV16 pseudovirion relative to w.t. L2**	**Co-assembly of L2 and encapsidation as for w.t**.	**RG-1 Mab Binding (WB)**	**% Binding of polyclonal 17-36 antiserum (ELISA)**
Δ17 - 30	>0.1%	Yes	No	-

Δ23 - 36	>0.1%	Yes	No	-

Δ353 - 362	100%	Yes	Yes	-

Δ393 - 403	100%	Yes	Yes	-

Y19A	100%	Yes	Yes	-

K20A	100%	Yes	No	100%

C22A	>0.1%	Yes	No	84%

C22S	>0.1%	Yes	No	96%

K23A	100%	Yes	Yes	-

Q24A	100%	Yes	Yes	-

C28A	>0.1%	Yes	No	74%

C28S	>0.1%	Yes	No	72%

P29A	100%	Yes	Weak	100%

C22/28S	>0.1%	Yes	-	120%

L1 alone	>0.1%	-	-	-

HPV16 pseudovirions were prepared using expression vectors for SEAP, HPV16 L1 and wild type L2 or the L2 deletion mutants or point mutants indicated. The preparations were treated with benzonase to remove unencapsidated DNA, and virions purified on an optiprep gradient. The purified virions were examined for L1 by Western blot (WB) with MAb885 antibody, or for L2 with RG-1 antibody or a full length HPV16L2-specific polyclonal antibody, or for encapsidated DNA by agarose gel-electrophoresis after extraction. The infectivity of 2-fold serially diluted pseudovirions was examined by measuring SEAP activity of 293TT cells three days after treatment with different volumes of the pseudovirion preparations. Rabbit polyclonal antiserum to HPV16 L2 17-36 peptide was tested for binding to HPV16 pseudovirions containing mutant L2 versus wild type L2 in an ELISA. (-) not done.

These findings suggested that the 17-36 region containing the RG-1 epitope is critical to viral infection whereas residues 353-362 and 393-403 are not. Therefore we applied alanine scanning mutagenesis to highly conserved amino acids within the 17-36 region to identify the contributions of individual residues to infection. Mutations Y19A, K20A, C22A, K23A, Q24A, C28A, or P29A were introduced into HPV16 L2. By comparison with wild type HPV16 L2, the introduction of these point mutations within L2 had no significant impact upon the yield of particles in the appropriate fraction, as demonstrated by L1 Western blot analysis (Table 1). Likewise, Western blot analysis of purified HPV16 pseudovirions revealed similar levels of wild type and point mutant HPV16 L2 were present (Table 1), suggesting that none of the point mutations within L2 adversely impacted L1/L2 co-assembly into HPV16 pseudovirion particles. However, the RG-1 monoclonal antibody did not react with K20A, C22A, C28A L2 by Western blot. Reaction with the P29A L2 mutant was weak, whereas the Y19A, K23A, and Q24A mutant L2 proteins were bound by RG-1 similarly to the wild type HPV16 L2 in Western blots. All of the L2 point mutant pseudovirions exhibited a wild type level of infectivity with the exception of the C22A and C28A mutants that lacked infectious activity upon 293TT cells using either a SEAP or GFP reporter (Table 1).

Since the thiol groups of cysteine residues 22 and 28 might be required for disulphide bridging or for hydrogen bonding, we tested the impact of C22S and C28S mutations upon both RG-1 binding and viral infectivity. Either of the C22S and C28S mutations prevented the recognition of HPV16 L2 by RG-1 in a Western blot. Like the C22A and C28A point mutants, HPV16 pseudovirions containing single C22S or C28S point mutations were not detectably infectious in vitro for 293TT cells using the SEAP assay. These results are consistent with those recently reported by Campos and Ozbun in 293TT cells and the spontaneously immortalized human keratinocyte cell line HaCaT [13]. Conversely, they differ from those of Conway *et al *who describe a 3-4 log increase in infectivity of HaCaT cells per encapsidated genome for L2 C22S or C28S containing HPV16 virions produced in the raft culture system [14]. This observation may reflect a reduced ability of the L2 C22S or C28S containing HPV16 particles to protect the encapsidated genome from sonication and benzonase treatment during measurement of encapsidated viral genome, resulting in very high specific infectivities of mutant as compared to wild type virions [14]. Neither study tested the impact of these mutations upon HPV16 infection of squamous epithelium [13,14].

**Figure 1:**
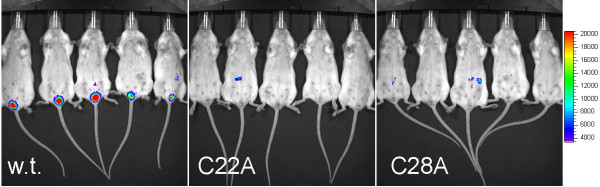
**Impact of L2 C22A and C28A point mutations upon vaginal infection of mice by HPV16 pseudovirions**. Mice (5/group) were treated with Depo-provera 4 days prior to challenge and then microtrauma was produced in the vaginal epithelium using a cytobrush [14]. The mice were then challenged intra-vaginally with equivalent doses, as determined by L1 concentration, of HPV16 pseudovirions containing a luciferase reporter plasmid and wild type L2 or single C22A or C28A point mutations, in a carboxymethylcellulose gel [2]. Three days post-challenge, luciferin was introduced into the vaginal vault and as a surrogate of infection the production of light by luciferase was visualized using a Xenogen IVIS 200 instrument.

A mouse model of HPV infection in the vaginal squamous epithelium has recently been developed as an alternative to tissue culture studies [5,15]. Therefore we explored the infectivity of the HPV16 pseudovirions containing single C22A or C28A point mutations as compared to wild type L2 in the mouse vaginal challenge model. As for the *in vitro *studies in human cell lines, HPV16 pseudovirions containing single C22A or C28A point mutations were dramatically less infectious than those containing wild type L2 (p < 0.01, for both mutants versus w.t.), but were not significantly different from each other (Figure 1).

It is possible that the point mutations of C22 or C28 affect either cell surface binding or appropriate trafficking of virions during infection. Campos and Ozbun observed indistinguishable cell surface binding and uptake into endosome/lysosomes co-incident with LAMP-1 at 8 h post infection [13]. Similarly we observed no clear differences in binding and trafficking between the wild type and the C22S or C28S mutant HPV16 pseudovirions as viewed at 2 h, 6 h or 20 h post infection by immunofluorescent staining of the virions with a rabbit polyclonal antiserum raised to HPV16 L1 VLP (not shown).

**Figure 2:**
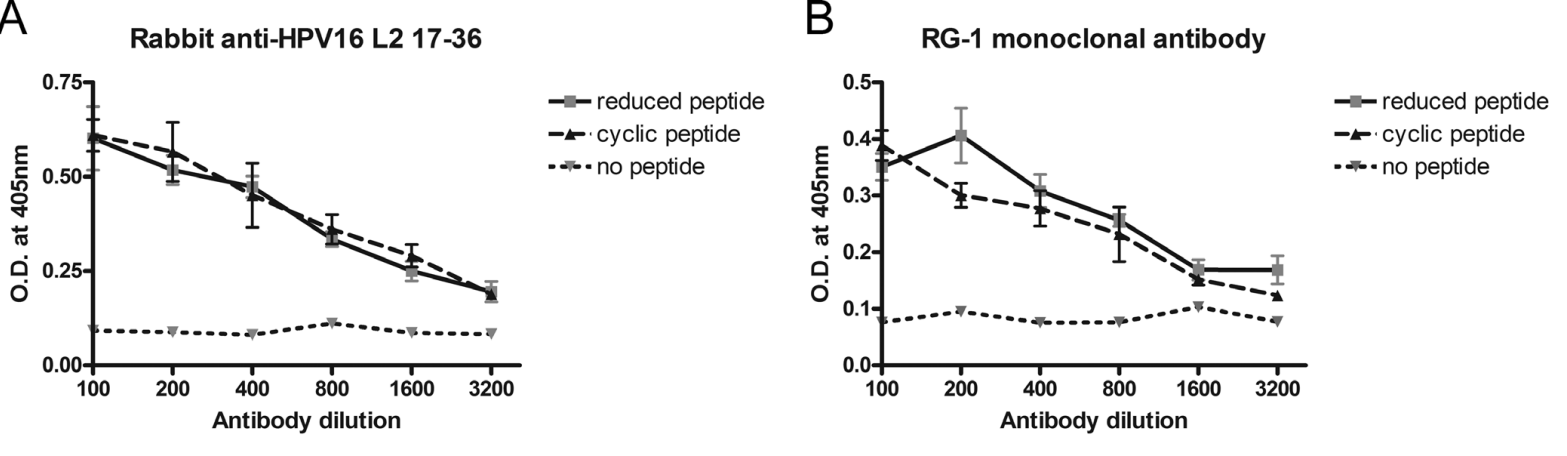
**Binding of RG-1 antibody to disulphide hairpin of HPV16 L2**. An HPV16 L2 13-31 cyclized peptide, purified to >99% homogeneity by HPLC, was completely reduced by the addition of 50 mM DTT and heating to 60°C. The redox state of the cyclized and reduced peptide was confirmed by mass spectrometry. The main neutral mass in the reduced sample was 2208.13Da and was 2Da larger than the 2206.13Da mass of the cyclized peptide. These main masses correspond to the expected masses of biotinylated HPV16 L2 13-31 peptide without and with disulfide bonds, respectively. The same day that the mass spectrometry was performed, the peptides were used to coat a microtiter plate. The binding of dilutions of RG-1, or a rabbit polyclonal antibody raised against HPV16 L2 17-36 peptide fused to KLH [2], to the cyclized or reduced HPV16 L2 13-31 peptide was then compared by ELISA (B) using peroxidase-linked anti-mouse or anti-rabbit IgG secondary antibodies respectively.

Campos and Ozbun also demonstrated that residues C22 and C28 form an intramolecular disulphide bond in HPV16 pseudovirions [13]. The presence of an L2 C22-C28 disulphide bond in HPV16 pseudovirions was surprising since the neutralizing monoclonal antibody RG-1 binds to reduced L2 in Western blot analysis [2]. Conway *et al *observed that HPV16 and HPV18 pseudovirions are resistant to neutralization by RG-1 early in raft culture production process, but they become sensitive as the rafts mature [14]. Since they also observe that the redox environment within the rafts and susceptibility to RG-1 neutralization changes over this period [16], it is possible that the redox state of C22 and C28 of L2 impacts their recognition by RG-1. Therefore we sought to determine whether RG-1 also binds to the L2 C22-C28 disulphide bonded epitope. To address this question we synthesized a biotinylated HPV16 L2 13-31 cyclized peptide and reduced peptide. The redox status of the cyclized and reduced peptide was confirmed by mass spectrometry, since the mass of the latter is 2Da greater. The RG-1 monoclonal antibody and a rabbit polyclonal antibody to HPV16 L2 17-36 peptide each reacted indistinguishably with the cyclized and reduced HPV16 L2 13-31 peptide in an ELISA (Figure 2). Therefore both RG-1 monoclonal antibody and the rabbit polyclonal antibody to HPV16 L2 17-36 peptide are able to bind to the L2 C22-C28 disulphide bonded or reduced HPV16 L2. This suggests that the differing sensitivity to RG-1 neutralization of HPV16 and HPV18 virions derived from 10- and 20-day organotypic raft cultures does not reflect a change in the redox state of the C22 to C28 disulphide bridge [14,16].

**Figure 3:**
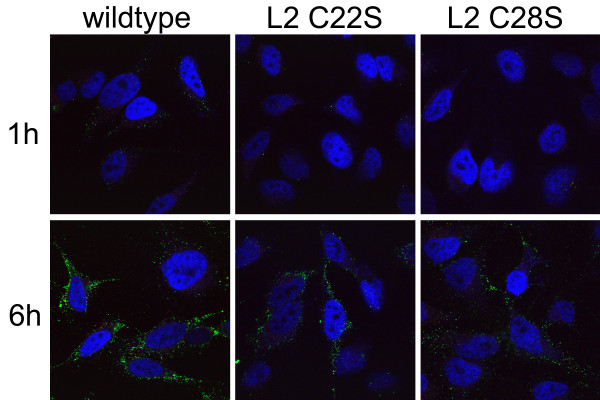
**Impact of L2 C22A and C28A point mutations upon display of residues 17-36 on the capsid surface**. HPV16 pseudovirions containing L2 with wild type or C22S or C28S mutations were bound to the surface of HeLa cells for one hour at 37°C. These cells were either fixed (1 h) or washed and chased at 37°C for five hours prior to fixation (6 h). The exposure of the HPV16 L2 17-36 epitope upon the virion surface was then determined by immunofluorescent staining with the rabbit polyclonal antibody to HPV16 L2 17-36 peptide, as previously described [1,4].

Furin recognizes residues 9-12 of HPV16 L2 and this cleavage removes 12 N-terminal amino acids from L2 and renders residues 17-36 accessible on the capsid surface for binding to RG-1 or the rabbit polyclonal antiserum to this epitope [1,4]. This process of revealing the 17-36 epitope upon virions takes several hours after their binding to the cell surface and is blocked by furin inhibitors or a subset of neutralizing monoclonal antibodies to L1. We hypothesized that the point mutations of L2 C22 or C28 to serine blocked infection by preventing the display the 17-36 epitope on the virus surface. To address this hypothesis, HPV16 pseudovirions containing L2 with wild type or C22S or C28S mutations were bound to HeLa cells for one hour, and then unattached virions were removed by washing and cells were incubated for an additional five hours at 37°C. As expected, there was minimal exposure of the RG-1 epitope after one hour, as determined by immunofluorescent staining with the rabbit polyclonal antibody to HPV16 L2 17-36 peptide (Figure 3). After the 5 h chase, there was robust staining of wild type HPV16 pseudovirions with the rabbit polyclonal antibody to HPV16 L2 17-36 peptide, consistent with the previously described conformational changes and furin cleavage to expose this epitope [4]. Surprisingly, the staining of the cells exposed to HPV16 pseudovirions containing L2 C22S or C28S mutations was only marginally weaker than that of the wild type particles (Figure 3). Similar findings were observed when infecting HaCaT cells (not shown). This likely reflects the slightly reduced (~30%) binding of HPV16 L2 17-36 peptide antiserum to L2 C22S or C28S (Table 1) rather than inefficient furin cleavage of the C22S or C28S mutant L2 in HPV16 pseudovirions. Thus these findings suggest that the C22 and C28 residues are not required for exposure of this epitope during HPV16 infection.

Our findings indicate that the neutralizing epitope within residues 17-36 contains cysteine residues that are critical for HPV16 pseudovirion infection and for neutralization by RG-1 [2]. These cysteine residues are not necessary for the initial binding and uptake of the virus. They are also not required for display of the 17-36 epitope on the virion surface, and are thus presumably dispensable for furin cleavage of L2 [1,4].

It is possible that these two cysteines, and potentially the neighboring conserved residues, are critical for binding to some cellular factor that mediates papillomavirus infection. However, mutation of L2 Y19A, K20A, K23A, Q24A, or P29A had no significant impact upon infectivity. Alternatively the disulphide hairpin between C22 and C28 might hold this region of L2 in a particular conformation, and that its reduction upon uptake acts as a molecular switch that is critical for infection, acting at a step following furin cleavage. Since such a mechanism might be common among papillomaviruses, we tested whether the equivalent two cysteine residues are also critical for infection by a highly divergent papillomavirus type, bovine papillomavirus type 1 (BPV1), which infects a different host at a cutaneous site and induces typically benign fibropapillomas. As observed for HPV16, the mutation of second cysteine residue in L2 to serine (C25S) dramatically reduced the infectivity of BPV1 pseudovirions produced in the 293TT cells and carrying the SEAP reporter to <0.1% of wild type [11]. Surprisingly, in contrast to the double mutation in HPV16 L2, the BPV1 L2 C19S, C25S double mutation reduced BPV1 pseudovirion infectivity of 293TT cells by only 50%. This latter finding suggests that the contribution of the disulphide bond between these two cysteine residues exerts subtle conformational changes within L2 during virion assembly that impact infectivity in diverse papillomavirus types. Further research to address potential differences in capsid protein conformation of HPV pseudovirions/quasivirions and organotypic raft and wart-derived virions during particle maturation is also warranted. Although it is unclear exactly how the L2 17-36 region contributes to papillomavirus infectivity, it remains an intriguing target for the development of broadly protective HPV vaccine [17].

## Abbreviations

HPV: human papillomavirus; BPV: bovine papillomavirus; SEAP: Secreted alkaline phosphatase; GFP: green fluorescent protein.

## Competing interests

RBSR is a paid consultant of Merck & Co, Inc., and Knobbe Martens Olson & Bear LLC. SJ and RBSR have received unrestricted educational grant funding from GlaxoSmithKline. RBSR, SJ and RG are co-inventors on L2 patents licensed to Shantha Biotechnics, Ltd., PaxVax, Inc. and Acambis, Inc. The terms of these arrangements are being managed by Johns Hopkins University in accordance with its conflict of interest policies.

## Authors' contributions

RG and SJ generated the mutant pseudovirions and tested their infectivity and reactivity with RG-1. BK performed ELISA studies with RG-1. PMD performed the microscopy experiments. RBSR conceived, designed and wrote the study.
